# N-acetylcysteine tiherapeutically protects against pulmonary fibrosis in a mouse model of silicosis

**DOI:** 10.1042/BSR20190681

**Published:** 2019-07-19

**Authors:** Huaping Huang, Mingjing Chen, Feng Liu, Haifeng Wu, Jie Wang, Jialiang Chen, Meihua Liu, Xi Li

**Affiliations:** 1 Department of Respiratory Diseases, The First Affiliated Hospital of Hainan Medical University, Haikou 570102, Hainan, China; 2 Department of Pathology, The First Affiliated Hospital of Hainan Medical University, Haikou 570102, Hainan, China

**Keywords:** anti-inflammation, antioxidation, N-acetylcysteine, pulmonary fibrosis, Silicosis

## Abstract

Silicosis is a lethal pneumoconiosis disease characterized by chronic lung inflammation and fibrosis. The present study was to explore the effect of against crystalline silica (CS)-induced pulmonary fibrosis. A total of 138 wild-type C57BL/6J mice were divided into control and experimental groups, and killed on month 0, 1, 2, 3, 4, and 5. Different doses of N-acetylcysteine (NAC) were gavaged to the mice after CS instillation to observe the effect of NAC on CS induced pulmonary fibrosis and inflammation. The pulmonary injury was evaluated with Hematoxylin and eosin/Masson staining. Reactive oxygen species level was analyzed by DCFH-DA labeling. Commercial ELISA kits were used to determine antioxidant activity (T-AOC, glutathione peroxidase (GSH-Px), and superoxide dismutase (SOD) and cytokines (TNF-α, IL-1β, IL-4, and IL-6). The expression of oxidising enzymes (NOX2, iNOS, SOD2, and XO) were detected by real time PCR. Immunohistochemistry (IHC) staining was performed to examine epithelial-mesenchymal transition-related markers. The mice treated with NAC presented markedly reduced CS-induced pulmonary injury and ameliorated CS-induced pulmonary fibrosis and inflammation. The level of malondialdehyde was reduced, while the activities of GSH-PX, SOD, and T-AOC were markedly enhanced by NAC. We also found the down-regulation of oxidising enzymes (NOX2, iNOS, SOD2, and XO) after NAC treatment. Moreover, E-cadherin expression was increased while vimentin and Cytochrome C expressions were decreased by NAC. These encouraging findings suggest that NAC exerts pulmonary protective effects in CS-induced pulmonary fibrosis and might be considered as a promising agent for the treatment of silicosis.

## Introduction

Silicosis is considered as one of the serious types of pneumoconiosis and a potentially fatal occupational fibrotic lung disease [1], which is highly prevalent in developing countries, especially in China, South Africa, and Brazil [2]. Accumulating evidence shows that silicosis is caused by long-term occupational or environmental exposure to free crystalline silica (CS) particles [3]. There are estimated to be tens of millions of workers exposed to CS worldwide and industrialization processes have made this situation even worse [4]. To date, the increasing mortality of silicosis has made silica exposure a high-priority public health concern in countries worldwide [5].

It has been accepted that the hallmarks of lung silicosis consists of massive inflammation and significant lung fibrosis [6,7]. When respirable CS particles are inhaled, their entry into alveoli induces oxidative stress through the formation of reactive oxygen (ROS) and nitrogen species due to the generation of siloxil radicals after crystalline-silica fracturing [8]. Therefore, it leads to extensive fibroblast proliferation and deposition of extracellular matrix (ECM) with the lungs and further triggers cytotoxicity, oxidative stress, pulmonary inflammation, and eventually silicosis [9]. In the meantime, the progression of lung fibrosis is accompanied with infiltration of inflammatory cells including eosinophils, neutrophils, and macrophages. Macrophages, for instance, can phagocyte silica particles and become activated to release a multitude of mediators, such as histamine and serotonin, which are retained in lung throughout inflammation and mediate cascade reaction leading to fibrotic response [10]. In addition, alveolar macrophages, as well as their produced TNF-α, interleukin (IL)-1β and IL-6 has been suggested to play a crucial role in inflammation, as a hallmark of exposure to silica [11]. Besides the aberrant induction of pulmonary inflammation, the intratracheal silica instillation leads to the enlargement of thoracic lymph nodes. The mitogenic responses to T cell receptor stimulation are apparently reduced in lymphocytes from silica-exposed lymph nodes with markedly increased activation-induced cell death, suggesting that silicotic lung apoptosis has been also implicated in the development of the initial inflammation [12]. During the process, Fas-L expression was inversely associated with mast cells, collagen/elastic deposition. As the progression of silicosis involves a fibrotic phase in which the ECM is deposited and lung parenchyma is remodeled, researches have been focussing on the intervening the inflammation processes and CS-induced fibrosis of silicosis [13]. Therefore, there is an urgent need to identify antioxidative and anti-inflammatory agents that might be promising for the prevention and treatment of silicosis.

N-acetylcysteine (NAC), a semi-essential amino acid with a thiol side chain, is the acetylated precursor of cysteine. It is clinically used to treat a wide variety of medical issues and conditions [14], such as acute respiratory distress syndrome, heavy metal-induced toxicity, and ameliorate certain psychiatric disorders [15] for its multiple beneficial properties, including enhancing bone regeneration, reducing post-surgical complications [16], and preventing mutagenic irradiation [17]. Furthermore, NAC has been reported to possess antioxidant properties by scavenging reactive oxygen species (ROS) and also inhibit the activity of cyclooxygenase-2 and membrane lipid peroxidation induced by inflammation [18,19]. Coombes et al. suggest that NAC has reno-protective activities in chronic kidney disease [20] and neuro-protective effect on spinal cord-injury [21]. Preliminary data in rat model revealed that oral treatment with high-dose NAC during early silica exposure can ameliorate the activity of proinflammatory cytokines, down-regulating ROS, and mitochondrial apoptosis signaling, thus attenuating subsequent lung fibrosis, suggesting the potential of NAC in the treatment for silica-induced lung fibrosis [22,23]. In the present study, we employed a mouse model of silicosis and different doses of NAC, in order to determine the potential pulmonary protective effects of NAC and the underlying mechanism.

## Methods

### Animal groups and treatments

Total 138 female wild-type C57BL/6J mice aged 6–8 weeks were purchased from the Experimental Animal Center of Hainan Medical College and randomly divided into the following groups: (1) blank control group (*n*=18)-animals without any treatment; (2) CS-induced model group (*n=*30); and (3) NAC-treated groups-animals were underwent CS exposed, and then administrated with NAC via gavage every day at a dose of 1.73 mg/20g (low-dose, *n=*30, NAC-L group), 3.46 mg/20g (moderate-dose, *n=*30, NAC-M group), or 5.19 mg/20g (high-dose, *n=*30, NAC-H group), respectively. For CS-induced model group, suspensions of CS (CAS: 7631-86-9, 2 μm, U.S. Silica Co., WV, Sigma–Aldrich) were prepared in normal saline (0.9% [w/v] NaCl). Each animal was received CS administration (2.5 mg suspended in 60 μl of saline) through intratracheal instillation and then administrated with saline via gavage every day, as previously described [24]. On months 0 (24 h), 1, 2, 3, 4, and 5 after treatment, animals were killed and harvested. For blank control group, three mice were used at every time point. Total five mice were used for analysis for the other experimental groups at every time point. The present study was performed in strict accordance with the recommendations from the Guide for the Care and Use of Laboratory Animals of the National Institutes of Health. All mouse experiments were approved by the Ethics Committee on Animal Research of the Hainan Medical College.

### Preparation of bronchoalveolar lavage fluid

Bronchoalveolar lavage fluid (BALF) was processed and collected as previously described [25]. Briefly, mice were intraperitoneally injected with 10% chloral hydrate for euthanasia. Immediately after euthanasia, the trachea of each mouse was exposed through a midline incision and cannulated with a sterile 22-gauge Abbocath-T catheter. Approximately 1.0 ml of BALF was retrieved per mouse, and BALF samples were kept on ice to avoid cell lysis.

### Serum and tissue sample collection

Mice in the above groups were killed and blood samples were obtained from abdominal aorta. After centrifuged at 1500 rpm for 10 min, serum was collected and stored at −80°C. The lung tissues were removed and immediately frozen in liquid nitrogen. Some of the lung tissues were fixed with 4% paraformaldehyde in phosphate-buffered saline for 24 h. The remaining lung tissues were frozen in liquid nitrogen for RNA detection or ELISA assay.

### ELISA assay

The mouse TNF-α ELISA Kit (CSB-E04741m, CUSABIO), mouse IL-1β (CSB-E08054m, CUSABIO), mouse IL-4 (CSB-E04634m, CUSABIO), and mouse IL-6 (CSB-E04639m) were used to determine the concentrations of TNF-α, IL-1β, IL-6, and IL-4 in plasma or BALF, respectively following the manufacturer’s instructions.

### Analysis of mRNA expression by RT-PCR

Total RNA was extracted from mice lung tissues with TRIzol reagent (TaKaRa Biotech), and cDNA was synthesized from 1 μg of total RNA using the Bestar qPCR RT Kit (DBI). RT-PCR was performed using SYBR^®^ Premix Ex Taq™ (TaKaRa) with Roche 480 using the following primers: inducible nitric oxide synthase (iNOS) (F: 5′-GTTCTCAGCCCAACAATACAAGA-3′ and R: 5′-GTGGACGGGTCGATGTCAC-3′); NADPH oxidase 1(NOX1) (F: 5′-CCTGATTCCTGTGTGTCGAAA-3′ and R: 5′-TTGGCTTCTTCTGTAGCGTTC-3′); NOX2 (F: 5′-GTATTGTGGGAGACTGGACG-3′ and R: 5′-ACAGACTTGAGAATGGAGGC-3′); NOX4 (F: 5′-GGTGTCTGCATGGTGGTGGTATT-3′ and R: 5′-CAGCCAGGAGGGTGAGTGTCTA-3′); SOD2 (F: 5′-CAGACCTGCCTTACGACTATGG-3′ and R: 5′-CTCGGTGGCGTTGAGATTGTT-3′); xanthine oxidase (XO) (F: 5′-ATGACGAGGACAACGGTAGAT-3′ and R: 5′-TCATACTTGGAGATCATCACGGT-3′); internal standard β-actin (F: 5′-CATTGCTGACAGGATGCAGA-3′ and R: 5′-CTGCTGGAAGGTGGACAGTGA-3′). The expression data were normalized to actin and quantitated using the Stratagene Mx3000P Real time PCR (Agilent, U.S.A.). Relative expression was calculated using 2^−ΔΔ*C*^_T_ method.

### H&E staining, IHC, and Masson’s trichrome staining

Morphological changes in the lung tissue were investigated using H&E staining. The tissues were embedded in paraffin and cut into 4-μm thick sections with a microtome. H&E was used to perform a H&E staining. As for IHC, antibodies against E-cadherin (Cell Signaling), Vimentin (Cell Signaling), and Cytochrome C (Abcam) were used to incubate slices (4 μm) overnight after deparaffinized. Afterward, the slices were incubated with HRP-conjugated secondary antibody followed by diaminobenzidine and hematoxylin staining orderly. As for Masson staining, slices (5 μm) were stained with Masson trichrome solutions. All the stained slices were then observed under a light microscope (BX51; Olympus Corporation).

### Oxidative stress analysis

Lung tissues of mice were homogenized with saline (1: 9) on ice, followed by centrifugation at 2500 rpm for 10 min at 4°C. The supernatant was used to determine the antioxidant activity including total antioxidative (T-AOC), glutathione peroxidase (GSH-Px), superoxide dismutase (SOD) and oxidative stress marker malondialdehyde (MDA) using commercial ELISA kits from Jiancheng Institute of Biotechnology (Nanjing, China) according to the manufacturer’s instruction.

### Flow cytometric ROS analysis

Production of ROS in blood was measured using fluorescent dye 2′, 7′-dichlorodihydrofluorescein diacetate (DCFH-DA, Beyotime Institute of Biotechnology, China) according to the manufacturer’s instruction. About 1 ml of blood was drawn from the main abdominal vein and anticoagulated with heparin. In brief, two test tubes were prepared, one without PMA (Phorbol 12-myristate 13-acetate, ROS trap phorbol), and the other with PMA, PMA (−) tube and PMA (+) tube. Anticoagulation with heparin 0.1 ml was added to each test tube. DCFH-DA 2 ml was added into the two tubes respectively and then placed in a 37°C water bath for 15 min. To prevent neutrophil agglutination, EDTA 500 μl was added into two tubes and then two tubes were placed in a 37°C water bath for 20 min. Supernatant was removed by centrifugation at 1500 rpm for 5 min. After being washed with PBS twice, cells were resuspended and fluorescence intensity of positive PMN (neutrophil) was measured by using flow cytometry (FACSCalibur, Becton–Dickinson).

### TUNEL assay

Fixed lungs were embedded in paraffin, and sequential 5-μm thick sections were processed for TUNEL staining using the ApopTag Peroxidase in Situ Apoptosis Detection Kit (Millipore, Billerica, MA, U.S.A.). Briefly, the lung tissue sections were stained and TUNEL-positive cells were observed under the microscope following the previous report [26].

### Statistical analysis

The presented data in the study were obtained in at least three independent experiments. These quantitative data were expressed as the mean ± S.D. with statistical analysis performed using the SPASS.19.0 statistics package (SPSS Inc., Chicago, U.S.A.). Differences between the groups were assessed by one-way ANOVA test. Statistical significance was set at *P* values < 0.05.

## Results

### Morphological findings of lung tissues in mice silicosis model

**Figure 1:**
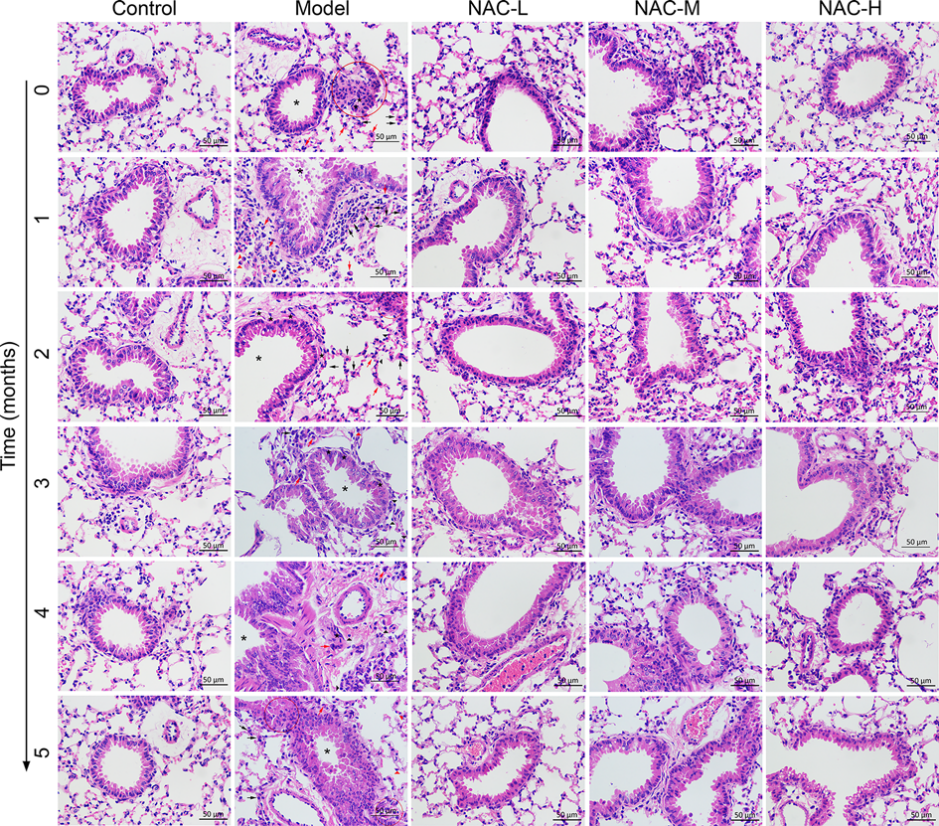
Effect of NAC on histological changes in lung tissue of in mice silicosis model stained with H&E (40× magnification). NAC can alleviate inflammatory infiltration in lung tissue compared with model mice. NAC-L, low-dose 1.73 mg/20 g of N-acetylcysteine; NAC-M, moderate-dose 3.46 mg/20 g of N-acetylcysteine; NAC-H, high-dose 5.19 mg/20 g of N-acetylcysteine. Asterisk indicated bronchial; black pentagon indicated spindle cells; red arrow indicated neutrophils; black arrow indicated macrophages (multinucleated, mononuclear, granular); red ring indicated tissue hyperplasia; red triangle indicated plasma cells; and black triangle indicated lymphocytes.

The morphometric assessment was performed in order to evaluate lung damage and inflammatory response in lungs from mice treated with CS. As shown in Figure 1, we found lung chronic inflammation, enlargement of the alveolar air spaces and destruction of the lung parenchyma in all CS-treated model mice, characterized by epithelial hyperplasia, granulomas and inflammatory cell infiltrates, compared with the normal alveolar architecture in blank control group. Moreover, time-dependent progression of the phenomenon was clearly observed. In particular, 2 months after silica administration, nodular silicotic granulomas accompanied by marked epithelial hyperplasia were found in central areas. After 4 months treatment, granulomas were confluent and included large acellular centers with peripheral infiltration of lymphocytes. To determine the effects of NAC on established mice silicosis model, NAC was administered after CS treatment. As expected, we observed an alleviation of emphysema in all NAC groups compared with the CS-induced model group. Moreover, moderate- and high-NAC treatment markedly reduced CS-induced pulmonary injury relative to the low-NAC group.

### The effects of NAC on oxidative stress in mice silicosis model

**Figure 2:**
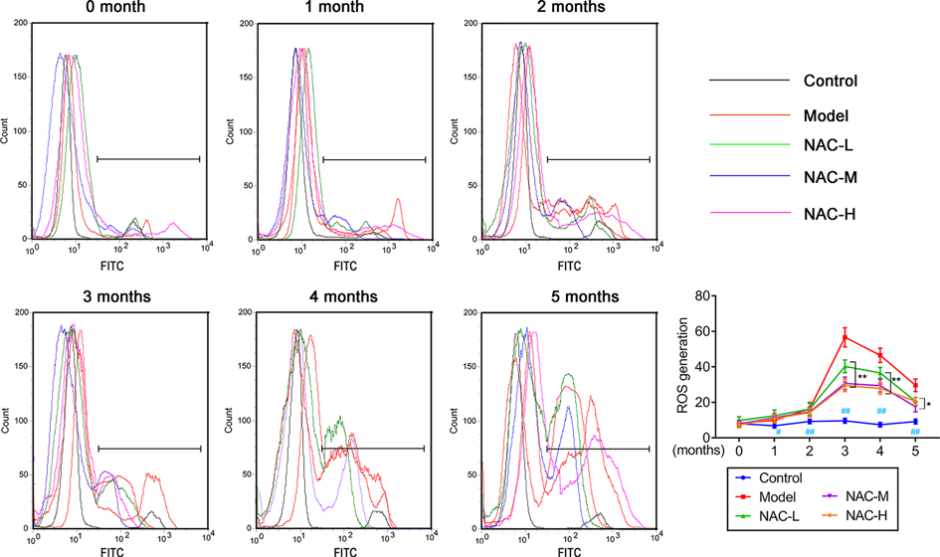
Effect of NAC on production of ROS in blood of in mice silicosis model using fluorescent dye 2′, 7′-dichlorodihydrofluorescein diacetate by flow cytometry NAC effectively decreased the ROS production compared with model in a dose-dependent manner (*n=*6).

To determine the CS-induced early-stage oxidative stress in mice, production of ROS in blood was measured with DCFH-DA assay. FACS analysis was performed. As shown in Figure 2, intracellular ROS nearly kept stable levels in blank control group. In model group, intracellular ROS significantly increased from 1 to 3 months, but slightly decreased at month 4 and 5. After pretreatment with NAC at low, moderate, and high concentration, respectively, intracellular ROS was significantly reduced compared with that in model groups, especially in 3, 4, and 5 months. Next, the levels of oxidative stress marker and antioxidants were determined using ELISA assay.

### NAC alleviated inflammatory response and oxidative stress through mediating expression of NOX1, NOX2, iNOS et al.

**Figure 3:**
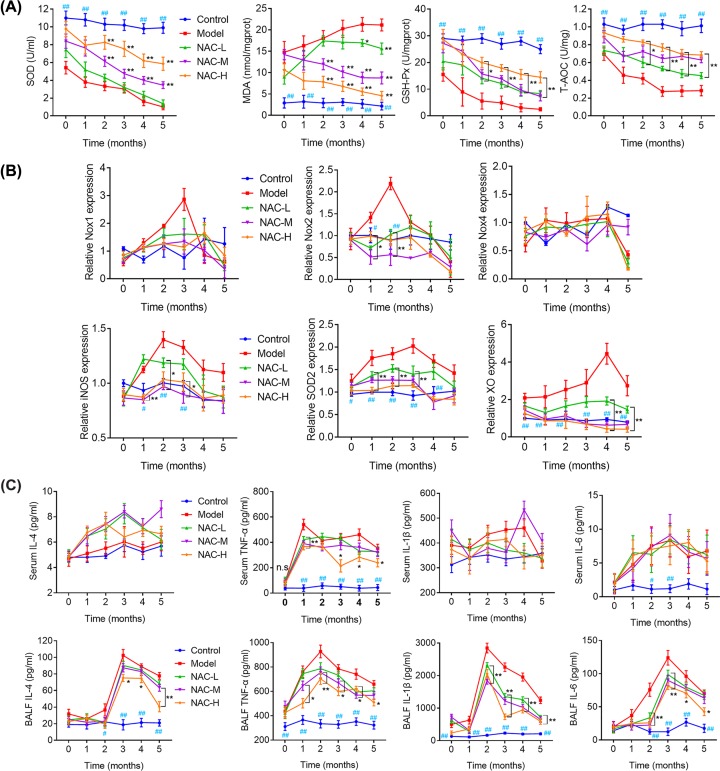
NAC affects oxidative stress and inflammatory response through mediating NOX1, NOX2, NOX4, iNOS, SOD2, and XO expression in mice silicosis model (**A**) Antioxidative effect of NAC in serum of mice silicosis model. (**B**) Expression of NOX1, NOX2, NOX4, iNOS, SOD2, and XO in response to NAC in lung tissues from each group using qRT-PCR analyses. (**C**) Inflammatory response of silicosis model mice to NAC in serum (upper panels) and BALF (lower panels). Data are expressed as mean ± S.E.M. ^#^*P<*0.05, ^##^*P<*0.01, *P<0.05, **P<0.01 versus model group.

As shown in Figure 3A, the oxidative stress marker, MDA was distinctly increased, but the levels of GSH-PX, SOD and T-AOC, the antioxidant markers to increase clarity, were reduced in the model group as compared with those in the control group. In contrast, mice from the NAC-treated groups recovered. Meanwhile, expression of NOX2, iNOS, SOD2, and XO mRNA increased in lung tissues after induced by CS compared with the control group in time-dependent manner. However, the tendency was reversed significantly by NAC administration compared with Model mice in a dose-dependent manner (Figure 3B).

To investigate the possible effect of NAC on inflammation, we measured the concentrations of TNF-α, IL-1β, IL-4, and IL-6 in plasma and BALF. As shown in Figure 3C, TNF-α was significantly elevated in plasma sample in model group compared with control mice but decreased by NAC administration. No significant differences in IL-1β, IL-6, IL-4, and IL-6 plasma levels were found between groups (Figure 3C upper panels). However, we found a significant increase of TNF-α, IL-1β, IL-4, and IL-6 in BALF from model group when compared with control group or NAC administration group (Figure 3C, lower panels). These results revealed that the administration of NAC alleviated the injury of airway inflammation and repressed pro-inflammatory cytokines in mice silicosis model.

### NAC administration alleviated the CS-induced tissue damages and pulmonary fibrogenesis in mice lungs

**Figure 4:**
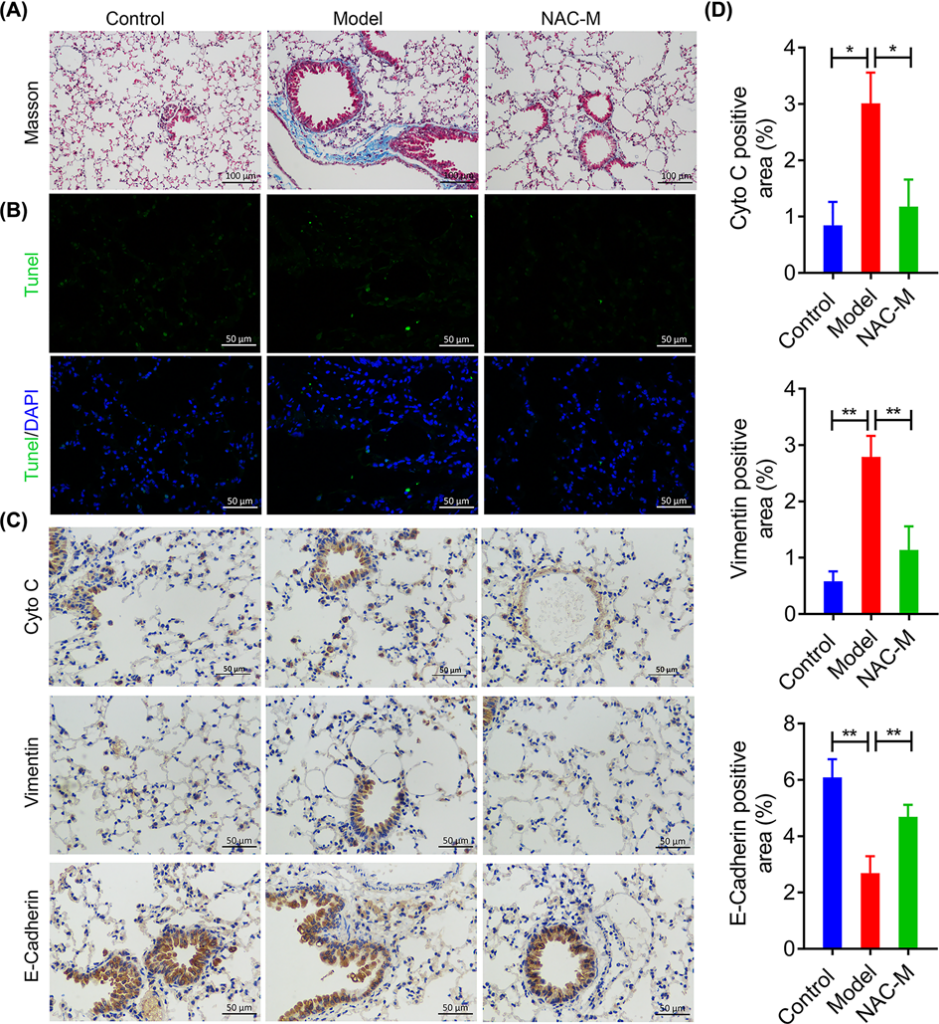
NAC administration alleviated the CS-induced tissue damages and pulmonary fibrosis in mice lungs (**A**) Representative images of Masson staining, measuring collagen deposition in lung tissues from CS-induced mice silicosis model following NAC administration for 5 months (20× magnification). (**B**) Representative images of TUNEL staining in the lungs tissues from CS-induced chronic pulmonary inflammation model mice following NAC administration for 5 months (40× magnification). (**C**-**D**) IHC analysis of the effect of NAC on the expression of Cyto C, E-cadherin and vimentin. *P<0.05, **P<0.01 versus model group.

To further evaluate the effects of NAC on CS-induced chronic pulmonary inflammation, we selected lung tissue samples of mice at the 5 months to perform pathological examination. Masson staining was first used to demonstrate silica-induced pulmonary fibrosis. As shown in Figure 4A, there were apparent increasing fibroblast and considerable fibroplasias (Blue), as substantial amount of collagen deposition (Red) was stained in model group compared with control group. These CS-induced symptoms were then obviously reversed by NAC administration. Moreover, TUNEL analysis showed that NAC can attenuate cell apoptosis induced by CS (Figure 4B). Collectively, NAC administration could partially alleviate the tissue damage and fibrogenic response in lungs from mice silicosis model.

Epithelial-mesenchymal transition (EMT) has been reported to play a key role in the pathologic process of lung injury and fibrotic lung diseases [27]. As shown in Figure 4C-D, E-cadherin was suppressed whereas vimentin was increased in lungs from mice following CS exposure. In contrary, NAC administration notably elevated E-cadherin expression but decreased vimentin and Cytochrome C expressions in CS-induced mice silicosis model. These results further indicate that CS exposure induces EMT characteristics in lungs from mice *in vivo*, which could be partially reversed by NAC administration.

## Discussion

Silicosis, as a type of chronic pulmonary diseases, is a progressive occupational lung disease caused by the long-term inhalation, deposition, and retention of CS particles [28]. In the present study, we established a mouse silicosis model by chronic exposure to CS in order to investigate the possible effects of NAC on the CS-induced chronic pulmonary disease. Interestingly, previous study with rat model adopted non-tracheal exposure method of disposable intrapulmonary injection of 50 g/l [22,23] while we ulitilized 42 g/l of CS for current study with mice model according to describe previously [24]. We found exacerbated lung injury, increased pulmonary inflammation, and severe fibrogenic response induced by CS exposure in model mice, which was in line with previous finding [29,30]. It suggests that chronic respiratory exposure to CS would increase lung tissue damages and fibrosis, even leading to the occurrence of lung cancer development. As silicosis is caused by long-term occupational or environmental exposure to free CS particles, we thus administered the highest dose of 5.19 mg/20 g (about 260 mg/kg) of NAC, which is much lower than 500 and 600 mg/kg, respectively [22,23], while we extended the observation duration from 28 days in earlier studies to over 5 months in order to simulate the pathogenesis of the disease with mice model. Notably, we observed that oral treatment of NAC in mice ameliorated CS-induced pulmonary fibrosis and inflammation. Consistent with our findings, increasing clinical and animal studies have reported that NAC could reduce acute inflammatory response and tissue damage to lung injury and sepsis, as well as improve impaired function [31–35].

Lung inflammation and fibrosis are the most common pathological changes resulting from inhalation of airborne particles. As the integral part of the pathogenesis of silicosis, inflammation was induced in the lungs by CS exposure, as demonstrated by elevating cytokines such as TNF-α, IL-1β, IL-4, and IL-6, which has been shown in the study of Li et al. [36]. Among these cytokines, TNF-α and IL-1β are recognized as the earliest factors in lung injury [37,38]. Consistent with the *in vivo* experiments, CS-injured mice given NAC had decreased levels of TNF-α, IL-1β, IL-4, and IL-6 in BALF.

Silicosis is also considered an oxidative stress related disease that can lead to the development of lung cancer [39]. Related studies have showed that phagocytes could produce oxidising enzymes leading to the generation of high levels of ROS during inflammation, which further promote inflammation in a feed forward loop manner [40,41]. Furthermore, oxidative damage caused by ROS may lead to various human diseases, such as cancer and inflammation [42]. As a major source of ROS, NOXs have a critical role in inflammatory response, contributing to ROS production during oxidative damage [43]. These facts further indicated the involvement of oxidative stress is related to chronic inflammation during exacerbation. To clarify the oxidative stress of NAC in mice, we investigated the levels of antioxidant enzymes, oxidative stress marker, and oxdizing enzymes. MDA is an indicator of oxidative stress that is inversely correlated with pulmonary function [44] and could result in the irreversible lung damage [45]. Related study has indicated that antioxidants, such as GSH-PX, T-AOC not only protect against the direct injurious effects of oxidants, but also change the inflammatory events involved in the chronic pulmonary diseases [45]. In our results, CS exposure distinctly increased the oxidative stress marker, MDA, but decreased the antioxidants (GSH-PX, SOD, and T-AOC). In addition, some oxidizing enzymes (iNOS, SOD2, and XO) were also significantly elevated in mice silicosis model. Correspondingly, we found that the antioxidants were increased and oxidizing enzymes were obviously decreased after NAC treatment. To confirm whether NOXs are related with silicosis, several NOXs, including NOX1, NOX2, and NOX4 were determined. The results demonstrated that elevated NOX2 levels in mice silicosis model were significantly reduced after administration with NAC, which suggested that NOXs might play an important role in lung inflammation and fibrosis.

In addition, we investigated the EMT characteristics in lungs from mice silicosis model *in vivo*. EMT is a process that polar adjacent epithelial cells transform to non-polar mesenchymal cells and increase cell mobility, which plays an important role in the development of pulmonary fibrosis [46]. According to the study of Liu et al., EMT was one of the key events in silica-induced pulmonary fibrosis [47]. Interestingly, EMT is closely related with the oxidative stress and its formation could be prevented by agents that have antioxidant properties, thus reducing pulmonary fibrosis [48]. Therefore, we were prompted to investigate whether NAC has a role in EMT characteristics *in vivo* by examining EMT-related markers in mice silicosis model using IHC staining. In line with expectations, we found a significant decrease of E-cadherin and increase of vimentin as well as Cytochrome C in mice silicosis model. On contrary, the expression of E-cadherin was strongly promoted, but vimentin expression was remarkably suppressed by NAC compared with those in the model group. The limitation in our study still exists that although we have tested various indicators, including T-AOC, GSH-Px, SOD, and oxidative stress marker MDA to determine antioxidant activity, hydroxyproline levels in lung tissue, as another indicator of oxidative stress, serum and BALF levels of IL-8 and high-sensitivity C-reactive protein ought to be assessed in further investigation,

N-Acetylcysteine (NAC) is a thiol-containing compound that has been used in clinical practice since the mid-1950s. It acts as a donor of cysteine, leading to replenishment of glutathione and also contributes to antioxidative and anti-inflammatory effects. NAC was originally introduced for the treatment of congestive and obstructive lung diseases. Accumulative data suggest that the drug itself does not accumulate in the body, but rather its oxidized forms and reduced and oxidized metabolites [49,50]. Recent evidence also indicated that, in adults with kidney impairment, NAC can be safely given both intravenous and intra-arterial at a dose of 450 mg/kg [51]. It has been demonstrated the effect of oral treatment with tetrandrine (TD) and NAC jointly on silica-exposed rats, which was better than single use of TD or NAC in alleviating SiO_2_-induced pulmonary fibrosis in rats [52]. Our further study may focus on the sustained or combinated use of NAC in the treatment of silicosis based on evaluation of toxicity and pharmacokinetics profile, which seeks to determine its feasibility in clinical practice.

**Figure 5:**
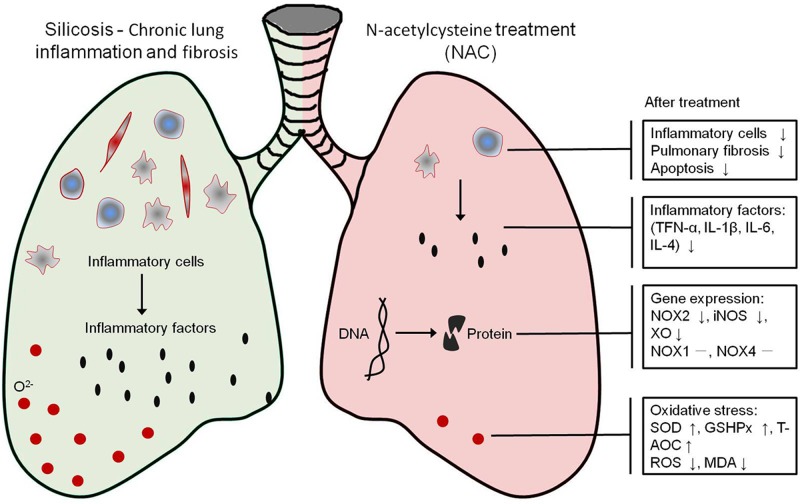
The mechanism of NAC treatment for CS-induced pulmonary fibrosis NAC treatment alleviated the CS-induced chronic lung inflammation and fibrosis. In mice silicosis model induced by CS, NAC treatment decreased number of inflammatory cells and inhibited pulmonary fibrosis and apoptosis, maybe through repressing the expression of NOX2, iNOS, XO, and attenuating oxidative stress.

In summary, our study demonstrated that NAC has protective effects against CS-induced pulmonary fibrosis partially via decreasing CS-induced pulmonary inflammation, ameliorating pulmonary fibrosis, and EMT-like characteristics (Figure 5). Based on these encouraging findings, NAC might become a promising agent for the treatment of silicosis in the future after more clinical investigations have been performed to determine whether NAC has a clinical application in CS-induced pulmonary fibrosis.

## Data Availability

The data used to support the findings of the present study are included within the article.

## Abbreviations

BALF: bronchoalveolar lavage fluid
CS: crystalline silica
ECM: extracellular matrix
EMT: epithelial-mesenchymal transition
GSH-Px: glutathione peroxidase
H&E: Hematoxylin & eosin
IHC: immunohistochemistry
MDA: malondialdehyde
NAC: N-acetylcysteine
iNOS: nitric oxide synthase
ROS: reactive oxygen species
SOD: superoxide dismutase
T-AOC: total antioxidative
TD: tetrandrine
